# Expression of the *SERPING1* gene is not regulated by promoter hypermethylation in peripheral blood mononuclear cells from patients with hereditary angioedema due to C1-inhibitor deficiency

**DOI:** 10.1186/s13023-014-0103-y

**Published:** 2014-07-22

**Authors:** Alberto López-Lera, Olga Pernia, Margarita López-Trascasa, Inmaculada Ibanez de Caceres

**Affiliations:** 1 Immunology Unit and IdIPAZ, University Hospital La Paz, Paseo Castellana 261, Madrid 28046, Spain; 2 Unit 754, Centre for Biomedical Research on Rare Diseases (CIBERER), Madrid, Spain; 3 Cancer Epigenetics Laboratory, INGEMM, University Hospital La Paz, Madrid, Spain; 4 Biomarkers and Experimental Therapeutics in Cancer, IdiPAZ, Madrid, Spain

**Keywords:** Promoter DNA methylation, C1-INH protein, SERPING1 gene, Hereditary Angioedema, PBMCs (Peripheral Blood Mononuclear Cells)

## Abstract

*SERPING1* mutations causing Hereditary Angioedema type I (HAE-I) due to C1-Inhibitor (C1-INH) deficiency display a dominant-negative effect usually resulting in protein levels far below the expected 50%. To further investigate mechanisms for its reduced expression, we analyzed the promoter DNA methylation status of SERPING1 and its influence on C1-INH expression. Global epigenetic reactivation correlated with C1-INH mRNA synthesis and protein secretion in Huh7 hepatoma cells. However, PBMCs extracted from controls, HAE-I and HAE-II patients presented identical methylation status of the *SERPING1* promoter when analyzed by bisulphite sequencing; the proximal CpG island (exon 2) is constitutively unmethylated, while the most distant one (5.7Kb upstream the transcriptional start site) is fully methylated. These results correlate with the methylation profile observed in Huh7 cells and indicate that there is not a direct epigenetic regulation of C1-INH expression in PBMCs specific for each HAE type. Other indirect modes of epigenetic regulation cannot be excluded.

## Background

C1-Inhibitor (C1-INH; OMIM#606860) is a SERPIN regulating the activation of the classical and lectin complement pathways, coagulation and fibrinolysis cascades [[1]]. It is mainly produced in the liver, although local synthesis by monocytes, fibroblasts and endothelial cells in inflammatory foci also exists. Deficiency of C1-INH due to mutations in the *SERPING1* gene causes Hereditary Angioedema (HAE) types I and II. HAE type I (HAE-I) can be due to mutations located throughout the gene sequence and is characterized by functional and antigenic C1-INH deficiency. HAE type II (HAE-II) is caused by point mutations affecting the reactive centre of the protein or nearby residues and presents with reduced function but normal antigenic levels of the protein. HAE patients suffer from recurrent disabling episodes of swelling in the subcutaneous and submucosal layers which can affect any body location and, if not promptly and properly treated, can lead to suffocation when the larynx is affected [[2]].

HAE is inherited in an autosomal dominant manner. Typically, HAE-I heterozygous patients exhibit C1-INH levels that are markedly below the expected 50% of controls, while type II patients have normal levels, but reduced function of C1-INH. Thus, regarding protein levels, HAE-I mutant alleles seem to exert dominant negative effects on their wild-type counterparts, rendering similarly reduced mRNA levels in both alleles to around 50% [[3]-[5]]. Additionally, a small percentage of HAE patients exhibiting reduced C1-INH function do not carry known *SERPING1* mutations even after exhaustive sequence analyses and MLPA screening; therefore, alternative modes of regulating C1-INH remain to be identified.

DNA methylation is an epigenetic event that plays a role in development, aging, chromatin structure, genomic imprinting and gene and microRNA expression. Altered DNA methylation patterns have been described in many genes and microRNAs associated with disease [[6]-[8]], including some members of the SERPIN superfamily, whose epigenetic alterations have been described in human placental diseases [[9]]. To contribute to the understanding of C1-INH's regulation of expression in HAE, we have investigated the CpG methylation status of the C1-INH locus and its surrounding regions in the hepatoma cell line Huh7 and in stored DNA samples from PBMCs obtained from healthy donors, HAE-I and HAE-II patients.

## Results and disussion

### Identification of putative CpG islands in the *SERPING1* locus

The screening for CpG islands was initially performed in the *SERPING1*’s genomic region containing its coding sequences (exons 1 to 8 and their adjacent introns). Next, we extended the search 6 kb in 5’ direction until the closest CpG cluster addressing the selection criteria:

More than 55% of GC dinucleotides (i), ObservedCpG vs. Expected CpG ratio: 0.65 (ii), Length: at least 200 bp (iii), Distance: 100 bp (iv).

**Figure 1:**
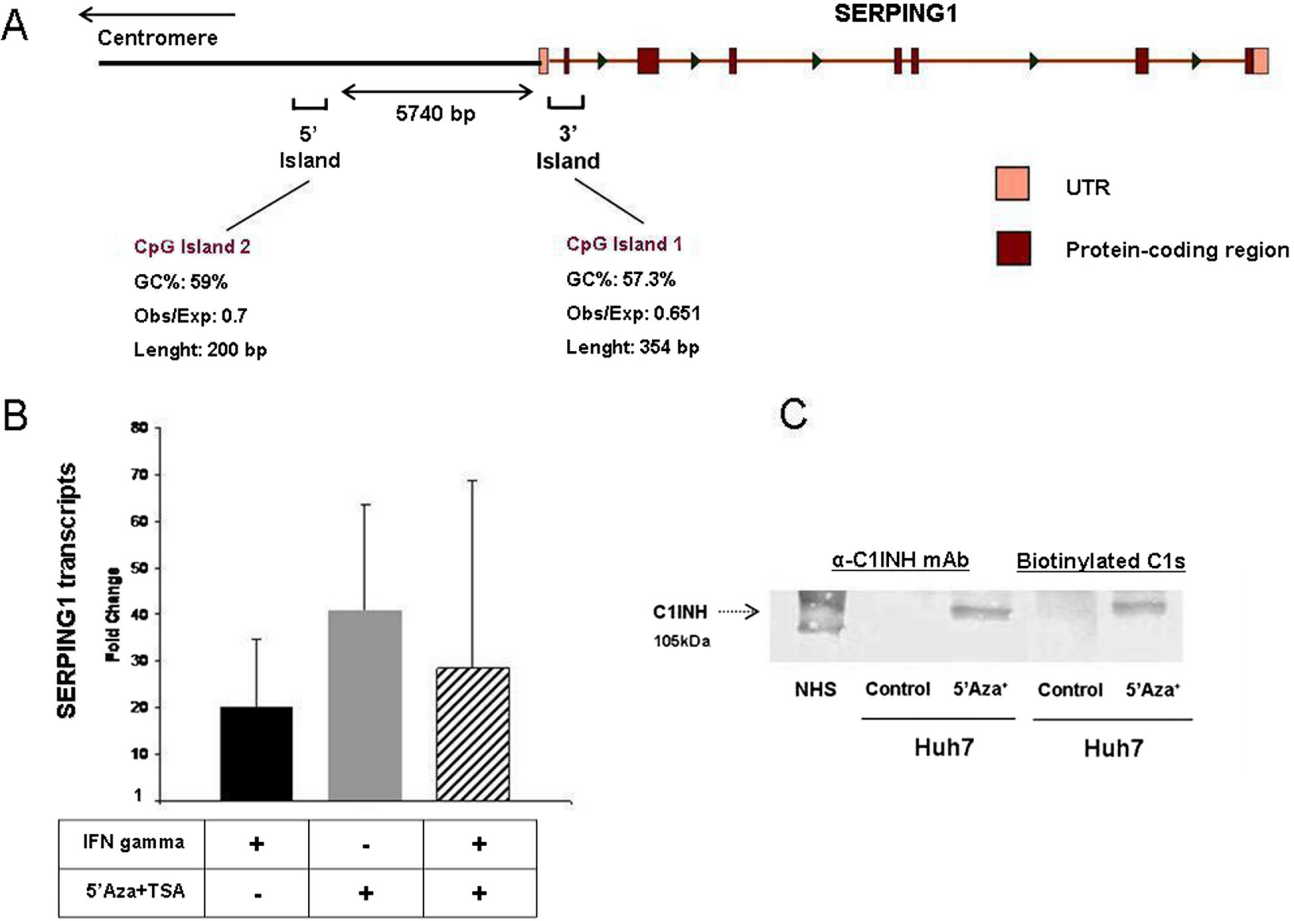
**Re-expression studies. (A)** Schema of the *SERPING1* locus indicating the positions of the most proximal CpG islands. CpG island 1 extends over exon 2 and comprises also part of the contiguous introns 1 and 2, while CpG island 2 is located 5740pb upstream exon 1. **(B)** Global demethylation induced by 5-Aza-2′-deoxycytidine (5’Aza) and Trichostatin A (TSA), upregulates *SERPING1* mRNA expression in Huh7 hepatocellular carcinoma cells more effectively than IFN-γ treatment **(C)** resulting in an increased C1-INH protein expression in Huh7 culture supernatants, as indicated by western blot analyses with both anti-C1-INH mAbs and biotinylated C1s. Relative quantification of *SERPING1* expression was performed using equal amounts of mRNA extracted from unstimulated Huh7 cultures as reference.

Two relaxed CpG islands endorsed by literature [[9]] were identified in the 11q11-q13.1 region according to the CpG Island Searcher software (http://cpgislands.usc.edu/) [[10]] (Figure 1A). The nearest CpG island to the ATG site (referred to as CpG island 1 or 3' island) comprises the 73 bp-exon 2 of the *SERPING1* gene and its surrounding intronic regions, 58 bp upstream and 224 bp downstream the second exon. It is 354 bp in length and contains 57.3% of CpG dinucleotides. The second CpG island - termed CpG island 2 or 5' island- is located between 5940 and 5740 bp upstream of the gene's first exon. It contains 59% CpGs and spans 200 bp.

### Global demethylation induced by 5’Aza and TSA upregulates *SERPING1* expression in Huh7 cells at the mRNA and protein levels

To our knowledge, no studies focused on quantifying C1-INH synthesis in Huh7 cells have been published, but the matter has been extensively addressed in other hepatocellular carcinoma cell lines, especially in the HepG2 line. HepG2 cells synthesize and secrete functional C1-INH and its expression is enhanced by IFN-γ administration in a dose-dependent manner [[11],[12]].

We quantified C1-INH mRNA from 1 μg of total RNA extracted from 75% confluent Huh-7 control cell cultures and compared it to that of Huh7 cells treated with 5’Aza + TSA for 3 culture days as described [[13]]. Reverse-transcription with oligo-dT primers (Qiagen, Valencia, CA) and quantification using a SYBR Green system (Applied Biosystems, Foster City, CA) were performed as previously described [[14]].

Similarly to what is seen in IFN-γ treated cells, global demethylation strongly correlated with enhanced *SERPING1* mRNA synthesis. Furthermore, mRNA synthesis was higher in 5’Aza + TSA-treated cultures (38-fold) than in those exposed to 250UI/mL of IFN-γ for 3 days (20-fold). Combined addition of 5Aza, TSA and IFN-γ during 3 culture days produced a non-additive effect resulting in a 28-fold change upregulation of *SERPING1* expression (Figure 1B). This probably reflects the consequences of global DNA/histones demethylation on the transcriptional/translational regulation of the IFNG gene (encoding human IFN-γ) or other loci involved in the transduction of IFN-γ signalling. In fact, DNA and histones’ epigenetic marks are known to play a role in transcriptional activation and silencing of IFN-γ signalling [[15]].

C1-INH protein presents two different isoforms in serum and plasma by western blot analysis: a 105KDa protein representing its native, non-cleaved conformation and an additional 96 kDa form corresponding to its cleaved, latent, non-functional conformation. In our hands, Huh7 cultures exhibit a single 105 kDa native band, which suggests the absence of activity involving those serum proteolytic pathways regulated by C1-INH (Figure 1C). Western blot analysis of culture supernatants confirmed *SERPING1* synthesis observed at the RNA level; no bands were observed in the supernatants of control Huh7 cells after three days of culture, while a single 105 kDa band corresponding to the native conformation of C1-INH was visible in treated Huh7 cells both by monoclonal anti-C1-INH antibody and biotinylated-C1s' detection systems (Figure 1C). Those results indicate a possible epigenetic regulation of *SERPING1* gene expression in the Huh7 hepatoma cells.

### CpG island 1 is unmethylated in HAE patients and controls while CpG island 2 is methylated

Bisulphite sequencing of stored genomic DNA samples obtained from HAE PBMCs (8 type I and 8 type II, Additional file 1), 10 healthy donor PBMCs and Huh7 cells was carried out as previously described [[16]] These studies received approval from the ethical committee of Hospital La Paz.

We included genomic DNAs from a homozygous-deficient patient carrying the R378C missense mutation in exon 7 and a heterozygous relative (samples 3 and 3b, respectively). Genetic and biochemical characterization of these patients has been previously described [[17]]. The T157fsX78 frameshift mutation in HAE-I patients 4, 4b and 4c and the exon 4 deletion in patients 1 and 6 are expected to profoundly reduce *SERPING1* mRNA levels due to the introduction of premature stop codons. In contrast, the remaining type I and type II mutations do not have obvious implications on translation or protein structure.

**Figure 2:**
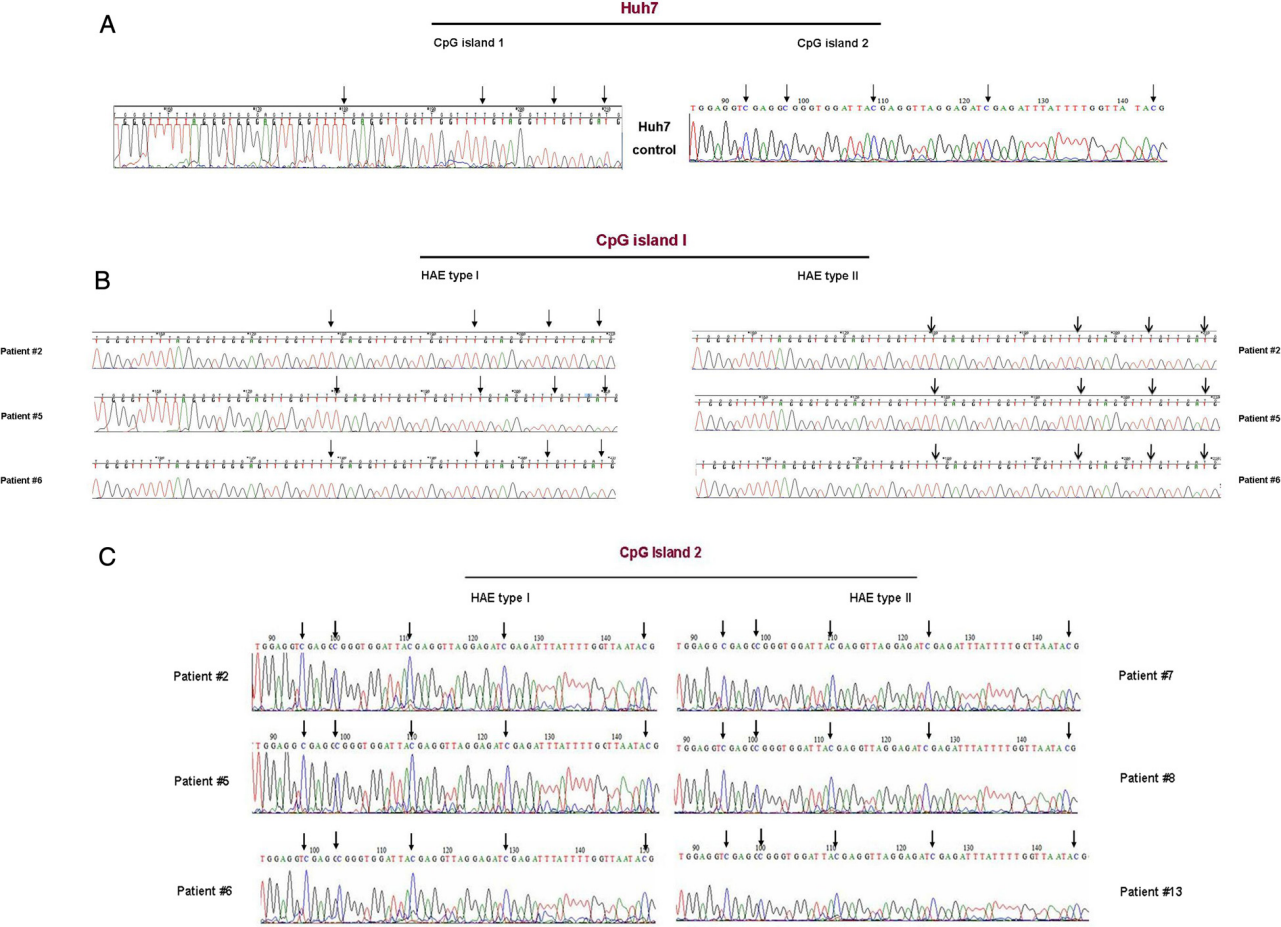
**Bisulphite sequencing.** Chromatograms showing the results of bisulphite sequencing in: **(A)** Huh7 hepatoma cells in CpG islands 1 and 2 (left and right panels, respectively), **(B)** Sequence of the bisulphite-modified CpG island 1 in type I and type II HAE patients (left and right panels, respectively) and (C) CpG island 2 type I and type II HAE patients (left and right panels, respectively) **(C)**. Arrows indicate the CpG positions susceptible of methylation. Wild Type sequences are: CpG Island 1: tgggctcccagggtgggagCtggctcCGaggctggctggctcCGcaggtcCGctgaCG. CpG Island 2: tggaggcCGaggCGggtggatcaCGaggtcaggagatCGagacccatcctggctaacaCG.

Sequencing results showed that in all cases the proximal CpG island 1 is unmethylated while the distal CpG island 2 is fully methylated (Figure 2). There is no difference between Huh7 cells, control PBMCs and those obtained from either HAE-I or HAE-II patients regarding the methylation status of both CpG islands 1 and 2 analyzed in this manuscript, indicating that there is no direct epigenetic regulation at DNA level. Therefore, transcriptional regulation of the *SERPING1* gene in pathological situations like HAE is not dependent on the methylation status of the analysed CpG islands. This epigenetic pattern is in agreement with that reported by Chelbi and collaborators in normal and preeclampsic placentas [[9]] and it is also concordant with repository data on DNase-I treatment and transcription factor binding site accessibility in the region, which shows a peak for DNase-I sensitivity centred in *SERPING1*'s exon 2 that matches CpG island 1 [[18]].

Considering the lack of evidence for differential epigenetic regulation at the DNA methylation level of promoter activity in the *SERPING1* locus in HAE patients’ PBMCs, the fact that the demethylating agents 5'Aza-dC and TSA strongly increased mRNA and protein synthesis in Huh7 cells could reflect an upstream indirect epigenetic regulation of trans-acting factors like transcription factors or microRNAs in the hepatocyte. Those elements could be reactivated by demethylation as has been reported in previous studies using similar experimental epigenetic reactivation approaches [[13],[19]]. On the other hand, we found no evidence for epigenetic mechanisms involving promoter CpG islands 1 and 2 playing a starring role in the negative dominance previously reported in HAE-I PBMCs.

The approach employed in this paper for the analysis of SERPING1 epigenetics is evidently hindered by the impossibility of obtaining hepatocyte samples from patients and controls in which to perform re-expression studies and bisulphite sequencing. Moreover, taking into consideration that monocytes do not undergo cell division, they are not suitable for 5’AZA + TSA re-expression studies, as a minimum of two cell divisions are required to completely remove epigenetic marks from DNA and histones. Therefore, these results are strictly limited to PBMCs and do not inform about epigenetic regulation in the hepatocyte, which is the main source of plasma C1-INH.

All considered, our data suggest that CpG island 1 is in a transcriptional-permissive chromatin conformation that would hypothetically allow the access of transcriptional machinery to the gene's promoter sites in PBMCs. The upregulating effect of demethylating agents on Huh7 cells would be due to epigenetic regulation of additional factors involved in *SERPING1*'s control of transcription and suggests an analogous mechanism could account for the differential C1-INH plasma levels characteristic of each HAE type.

## Competing interests

The authors declare that they have no competing interests.

## Authors’ information

López-Trascasa Margarita and Ibanez deCaceres Inmaculada are Senior authors.

## Additional file

## Supplementary Material

Additional file 1: Table S1 Patient cohort. Description of the 16 HAE patients studied. Patient referenced 3* carries the R378C mutation in homozygosis, while his brother referenced 3b* is an asymptomatic R378C heterozygote with a HAE type II biochemical profile. C1Fun: Function of C1-INH in the patient’s plasma expressed as a percentage of a healthy donor. (Normality range > 50%) (DOC 50 kb) (DOC 50 kb) Click here for file
