# Supplementary material for: Expression of the SERPING1 gene is not regulated by promoter hypermethylation in peripheral blood mononuclear cells from patients with hereditary angioedema due to C1-inhibitor deficiency
Source: Orphanet J Rare Dis. 2014 Jul 22;9:103. doi: 10.1186/s13023-014-0103-y (PMC4115163; doi:10.1186/s13023-014-0103-y)
Supplement: Additional file 1: Table S1 — Patient cohort. Description of the 16 HAE patients studied. Patient referenced 3* carries the R378C mutation in homozygosis, while his brother referenced 3b* is an asymptomatic R378C heterozygote with a HAE type II biochemical profile. C1Fun: Function of C1-INH in the patient’s plasma expressed as a percentage of a healthy donor. (Normality range > 50%) (DOC 50 kb) (DOC 50 kb) [file s13023-014-0103-y-S1.doc]

| **Patient** | **HAE type** | **Mutation** | **Gender** | **Age (years)** | **[C1-INH] mg/dL** | **C1Fun (%)** |
| --- | --- | --- | --- | --- | --- | --- |
| **1** |  | delEx4 | F | 54 | 3.99 | 13 |
| **2** |  | P482R | F | 73 | 3.37 | 10 |
| **3*** | **I** | R378C | M | 38 | 4.38 | 11 |
| **4** |  | T157fsX78 | F | 32 | <2.83 | 8 |
| **4b** |  | T157fsX78 | F | 22 | 4.14 | 6 |
| **4c** |  | T157fsX78 | M | 13 | 3.53 | 7 |
| **5** |  | A123D | F | 61 | <2.83 | 15 |
| **6** |  | delEx4 | M | 33 | <2.83 | 6 |
| **7** |  | I440S | M | 68 | 29 | 34 |
| **3b*** |  | R378C | M | 33 | 24.9 | 44 |
| **8** | **II** | R444C | M | 45 | 33.1 | 22 |
| **9** |  | R444C | F | 43 | 56.9 | 18 |
| **10** |  | R444C | M | 67 | 69.4 | 22 |
| **11** |  | R444C | F | 55 | 76.9 | 20 |
| **12** |  | R444C | M | 12 | 83.8 | 20 |
| **13** |  | R444G | M | 66 | 68.1 | 49 |
